# Whose Mind Matters More—The Agent or the Artist? An Investigation of Ethical and Aesthetic Evaluations

**DOI:** 10.1371/journal.pone.0070759

**Published:** 2013-09-11

**Authors:** Angelina Hawley-Dolan, Liane Young

**Affiliations:** Department of Psychology, Boston College, Chestnut Hill, Massachusetts, United States of America; Ecole Normale Supérieure, France

## Abstract

Theory of mind, the capacity for reasoning about mental states such as beliefs and intentions, represents a critical input to ethical and aesthetic evaluations. Did the agent cause harm *on purpose*? Were those brushstrokes *intentional*? The current study investigates theory of mind for moral and artistic judgments within the same paradigm. In particular, we target the role of intent for two kinds of judgments: “objective” judgments of quality and “subjective” judgments of preference or liking. First, we show that intent matters more for objective versus subjective judgments in the case of ethics and aesthetics. Second, we show that, overall, intent matters more for ethical versus aesthetic evaluations. These findings suggest that an “objective-subjective” dimension describes judgments across both domains, and that observers assign more weight to the mind of the moral agent than the mind of the artist when making the relevant evaluations.

## Introduction

Theory of mind, the capacity for reasoning about mental states such as beliefs and intentions, is a key cognitive process across a number of contexts, including moral judgment [1]]–[[3] and artistic evaluation [4]]–[[6]. People consider the harmful or helpful intentions of their social partners and not simply the outcomes of their actions [2]. Similarly, when evaluating a work of art, people consider the artist's mental state, i.e. what the artist planned or intended [7]]–[[9], [6]. Here, we compare the role of mental states in people's moral judgments of agents versus artistic judgments of artwork.

The key role of mental states in the domain of art has been explored in prior work [4], [10]. For example, how children name a picture depends on whether it was created intentionally or accidentally [10]. More broadly, people consider an object's origin when reasoning about its purpose [11] and when inferring ownership [12]. People also value authentic images made by professionals (e.g., Picasso) over duplicates [13], [7], [14].

Recent evidence suggests that people are even more likely to consider the artist's identity when they are instructed to deliver a judgment of the *objective* value or quality of the art versus a judgment of their own *subjective* preference or liking of the art [6]. In particular, participants viewed paired images in which one image was created by a professional artist and the other image by a child, chimpanzee, or elephant. When participants were asked for a subjective judgment, i.e. what they “liked”, they delivered a more subjective response (based on their own taste); participants also focused less on the process of creation and more on the end product [6]. By contrast, when asked for an objective judgment, i.e. what they thought was “better” art, participants delivered a more objective response (based on the compositional properties of the art); participants focused more on the process of creation (e.g., the artist's plans and intentions) than on the end product (e.g., the paint on the canvas). Structural models support these findings, suggesting that subjective preferences are based more on what is intuitively visually appealing, whereas objective evaluative judgments rely on more abstract principled reasoning [15].

The current study builds directly on this prior work by systematically investigating adults' subjective and objective judgments of intentionally versus accidentally created art. In particular, the current study seeks to determine (using the methodological approach of previous studies) whether objective evaluations (e.g., *is the art good?*) are more sensitive to perceived levels of intentionality—whether the work of art was created intentionally or accidentally—than subjective evaluations (e.g., *do you like the art?*). Put plainly, does theory of mind play a greater role in objective versus subjective evaluations of art? Do people perceive a greater “intentional versus accidental difference” when delivering objective versus subjective judgments?

A parallel body of work suggests that moral judgments also depend critically on theory of mind (see [16], [17] for recent reviews): people assign more blame for intentionally versus accidentally harmful actions and more praise for intentionally versus accidentally helpful actions [2], [18]. Recent research also shows that brain regions involved in theory of mind are robustly recruited for moral judgment [e.g., 19–23], and their disruption leads to a reduced role for intent in moral judgment [24]. Less is known, however, about the possible distinction between “subjective” versus “objective” moral judgments. Here, we determine whether, in the moral domain, objective judgments of moral agents (e.g., *is the agent a good person?*) versus subjective judgments (e.g., *do you like the agent?*), designed specifically to parallel the objective versus subjective aesthetic judgments, depend differentially on information about intent (e.g., “intentional versus accidental difference”).

Prior research has uncovered individual differences in “moral objectivism” versus “moral subjectivism”. This research suggests that some people are “moral objectivists”, taking ethical beliefs to express objective factual truths, whereas others are “moral subjectivists”, treating moral values more like subjective preferences [25], [26]]–[[28]. Note though that this prior work has focused on participants' *meta-ethical* intuitions about whether morality is objective or subjective (e.g., whether there are objectively right or wrong answers to moral questions or whether moral judgments simply reflect subjective preferences), whereas in the current work we are interested in participants' intuitive responses to questions about moral agents in a specific instance – *is this agent a morally good person* (*objective* evaluation), versus *do you like this agent* (*subjective* preference).

Nevertheless, some of this prior research indicates that the domain of morality, on the whole, may be viewed as more “objective” than the domain of art. In particular, moral codes are often perceived as universal across time and space and non-negotiable [29] as well as highly sensitive to information about agents' intent (e.g., [2]). By contrast, artistic preferences are sometimes described as similar to gustatory preferences (e.g., preferring vanilla over chocolate; [30]). Importantly, though, recent work discussed above has revealed that objective “value” judgments of *art* are in fact sensitive to the feature of intentionality [6]; indeed, a substantial body of work indicates the objective nature of aesthetic judgments [31]]–[[33]. Given this richly textured theoretical backdrop, one aim of the present study is to provide a systematic comparison of artistic and moral judgments on the same dimensions.

We hypothesize that if moral judgments are truly more “objective” than artistic judgments, then moral judgments on the whole may be more sensitive to intentionality than artistic judgments, just as objective judgments in both domains may be more sensitive to intentionality than subjective judgments. Note that in offering this hypothesis we do not deny the objective aspects of artistic judgments; instead, we wish to compare, using the same cognitive measures, artistic judgments to moral judgments. In particular, for both domains, we wish to investigate the difference between objective and subjective judgments and the critical “intentional versus accidental difference” discussed above.

The current study investigates the distinction between subjective versus objective judgments for art and morality, and the role of intent, or theory of mind, across both domains. At the broadest level, we hypothesize that aesthetic and moral judgments both rely on the shared cognitive process of theory of mind. We hypothesize further that (1) that intent matters more for objective versus subjective judgments across both domains and (2) overall, intent matters more for moral versus artistic judgments.

To test these hypotheses, we conducted a series of analyses. In Experiment 1, we conducted three ANOVAs (and associated paired samples *t*-tests) of participants' aesthetic judgments of intentionally and accidentally made artwork and participants' moral judgments of intentionally and accidentally performed negative (harmful) and positive (helpful) actions. First, we conducted a Judgment (objective vs. subjective) by Intent (intentional vs. accidental) ANOVA of participants' artistic judgments to measure the impact of intent on subjective versus objective artistic judgments. We hypothesized that intent would matter more for objective than subjective judgments (i.e., Judgment×Intent interaction). Second, we conducted a Judgment (objective vs. subjective) by Valence (positive vs. negative) ANOVA of participants' moral judgments, using a difference score measure of the intentional versus accidental difference; this analysis was aimed at measuring the impact of intent on subjective versus objective judgments of negative versus positive scenarios. Difference scores reflected the following subtraction: Judgment of Intentional stimuli minus Judgment of Accidental stimuli. Absolute values of difference scores (reflecting the *magnitude* of the effect of intent on judgments) were used in all analyses that included responses to positive and negative stimuli. We hypothesized that intent would matter more for objective than subjective judgments (i.e., main effect of Judgment). Third, we conducted a Judgment (objective vs. subjective) by Domain (art vs. morality) ANOVA of both artistic and moral judgments, using the same difference score measures above to determine both whether intent matters more for objective versus subjective judgments (i.e., main effect of Judgment) and whether intent matters more for moral versus aesthetic judgments (i.e., main effect of Domain).

In Experiment 2, we analyzed participants' aesthetic judgments of intentionally and accidentally made artwork (good and bad) and participants' moral judgments of intentional and accidental actions (positive and negative). First, we conducted paired samples *t*-tests for the domains of Art and Morality separately, using the same difference score measures above, e.g., is the intentional versus accidental difference greater for objective versus subjective judgments in both domains? Second, we conducted a Judgment (objective vs. subjective) by Domain (art vs. morality) by Valence (positive/good vs. negative/bad) ANOVA again to determine support for our primary hypotheses, i.e., whether intent matters more for objective versus subjective judgments and whether intent matters more for moral versus aesthetic judgments.

## Experiment 1

### 2.1. Participants

Participants were 31 undergraduate psychology majors at Boston College, who participated for course credit (11 males, 20 females, ages 18–27 years, *M* = 19.4).

*Ethics Statement:* Studies were approved by the Boston College Institutional Review Board, and written consent was obtained from each participant.

### 2.2. Materials and Procedure

Order of presentation of art and moral tasks was counterbalanced across participants.

#### Art

Stimuli consisted of four works of abstract art, selected from art history textbooks (e.g., Hans Hoffman). Images were presented one at a time and approximately equated in size and resolution. Frames and signatures were removed.

**Figure 1:**
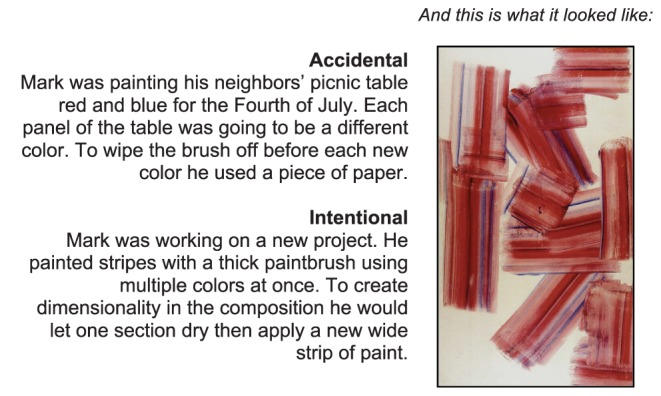
Sample “Good” Art Image, with narratives. Figure 1 consists of a sample of the stimuli used. The sample is an artwork belonging to the category of “good” art. The image is accompanied by the intentional and accidental narratives.

A brief narrative accompanied each image, describing how it was created and by whom. We constructed two versions of each narrative: accidental and intentional (Figure 1). Word count was matched across conditions. Each participant saw only one version for each image. (The full set of images and scenarios is available via Text S1.) Images were seen as accidental or intentional an equal number of times, across participants. These same conditions also applied to the morality component of the experiment.

Following the presentation of each story and image, participants delivered two kinds of judgments: (1) Subjective: “How much do you like this image at this moment?” (1 = Not at all, 7 = Very Much); (2) Objective: “Is this a good work of art?” (1 = No, absolutely not, 7 = Yes, absolutely). We included the phrase “at this moment” for subjective judgments, which are thought to change over time. By contrast, objective judgments are meant to express truths that are relatively stable over time. We also acknowledge that a rating of how “good” an entity is may sometimes reflect a subjective evaluation (e.g., “how good is the pie”). Importantly, though, our results as well as prior results [6] reveal the predicted systematic differences (i.e., the influence of intent) between subjective and objective judgments as elicited by the questions as phrased. Furthermore, these precise questions were used by Hawley-Dolan and Winner [6] to probe subjective versus objective judgments in the domain of art; importantly, this work revealed distinct cognitive signatures for subjective versus objective judgments, i.e., information about the artist's identity and objective compositional properties influenced *objective* judgments, whereas *subjective* judgments were driven primarily by participants' individual preferences.

#### Morality

**Figure 2:**
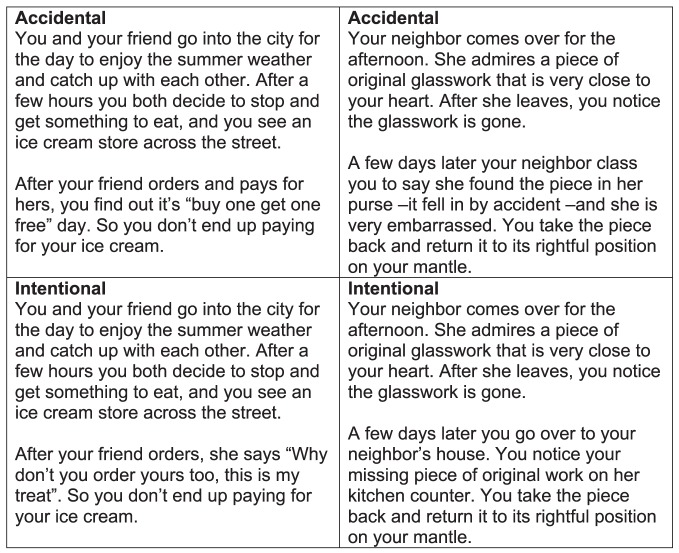
Sample Moral Scenarios. Positive moral stories on left, negative moral stories on right. Figure 2 consists of samples of the moral scenarios used. It is arranged by intentionality (intentional vs. accidental). The positive moral stories are located on the left, and the negative ones on the right.

Four positive scenarios described helpful actions; four negative scenarios described harmful actions. (The full set of moral scenarios is available via Text S1.) As above, we constructed two versions of each scenario: accidental and intentional (Figure 2). Following the presentation of each story, participants delivered two kinds of judgments targeting the moral agent: (1) Subjective: “How much do you like your [cousin, friend, etc.] at this moment? (1 = Not at all, 7 = Very Much); (2) Objective: “Is your [cousin, friend, etc.] a good person?” (1 = No, absolutely not, 7 = Yes, absolutely). We note that works of art and moral acts may both be experienced. For example, when an artist creates a work of art, the viewer typically makes a spontaneous judgment about the art – e.g., is the art good (objective), do I like it (subjective). The viewer may or may not evaluate the artist – e.g., is the artist good, do I like the artist. The art in front of the viewer (rather than the artist behind the scenes) may be more salient and more readily elicit a more spontaneous evaluation. Thus, we focus here on people's intuitive evaluations of the artwork (rather than the artist). By contrast, when a moral agent helps or harms a person, this person may spontaneously judge the agent – e.g., is the agent good (objective), do I like the agent (subjective) – in order to maintain or dissolve a relationship with the helpful or harmful agent. Thus, we focus here on people's intuitive judgments of the person (rather than the person's act – is the act good, do I like the act). In sum, to capture naturalistic intuitions for each independent domain, we elected to elicit judgments of the *art* in the case of art and judgments of *agents* in the case of morality.

To ensure that negative and positive stories were matched in severity or significance, a separate group of participants responded to two questions: (1) Is this a big deal to you? (1 = Not a big deal, 7 = A very big deal), and (2) Objectively speaking, is this event significant? (1 = Not at all, 7 = Very Much). Participants did not distinguish between negative and positive moral stories on either question (big deal: *t*(3) = −.375, *p* = .73; significance; *t*(3) = .176, *p* = .87).

### 2.3. Calculating Difference Scores for Data Analysis

To measure the impact of intent, that is, the perceived difference between intentional versus accidental actions, across *negative and positive* scenarios and art images, we calculated *difference scores*, as follows::: Judgment of Intentional [action or image] minus Judgment of Accidental [action or image], labeled below as IA (i.e., Intentional minus Accidental) difference scores. These difference scores were calculated separately for objective judgments and subjective judgments. Absolute values of IA difference scores were used where specified in Experiment 1 (i.e., when comparing across moral judgments of positive and negative items and when comparing moral judgments of these positive and negative items to aesthetic judgments of artwork) and in Experiment 2 throughout. In sum, the absolute values of IA difference scores reflect the *magnitude* of the effect of intent on judgments, critical for comparing participants' responses across negative and positive items.

## Experiment 1 Results

### 3.1. Art

A Judgment (objective vs. subjective) by Intent (intentional vs. accidental) ANOVA of participants' artistic evaluations revealed a significant interaction (*F*(1,30) = 6.12, MSE = 2.33, *p*<.019). In other words, intent (the intentional versus accidental difference) mattered more for objective than subjective judgments, as predicted. Paired samples *t*-tests revealed a significant difference due to intent for objective judgments (*t*(30) = 3.18, *p*<.03); that is, accidental images were judged to be worse (*M* = 3.7) than intentional images (*M* = 4.5). This difference was not significant for subjective judgments (*t*(30) = 1.12, *p* = .29); that is, accidental images (*M* = 4.1) were not judged worse than intentional images (*M* = 4.4).

### 3.2. Morality

The key analysis of participants' moral judgments was a Judgment (objective vs. subjective) by Valence (positive vs. negative) ANOVA of the absolute values of IA (i.e., Intentional minus Accidental) difference scores. It is important to note again that the absolute values of IA difference scores were analyzed rather than participants' raw moral judgments because participants judged *negative and positive* moral stories (in which agents acted intentionally and accidentally in harmful and helpful ways) on the same measures, i.e., (1) Subjective: “How much do you like your [cousin, friend, etc.] at this moment? (1 = Not at all, 7 = Very Much); (2) Objective: “Is your [cousin, friend, etc.] a good person?” (1 = No, absolutely not, 7 = Yes, absolutely). This analysis approach therefore allows us to target the *magnitude* of the effect of intent (i.e., the intentional versus accidental difference), the primary effect of interest, on moral judgments across negative and positive moral items. The ANOVA of the absolute values of IA difference scores revealed a main effect of Judgment (*F*(1,30) = 13.41, MSE = 13.55, *p*<.001). In other words, the intentional versus accidental difference was greater for objective than subjective judgments, as predicted. Paired-samples *t*-tests revealed that intent mattered more for objective than subjective judgments, for both positive (*t*(30) = −3.05, *p*<.005) and negative scenarios (*t*(30) = 3.42, *p*<.002).

The same Judgment (objective vs. subjective) by Valence (positive vs. negative) ANOVA also revealed a main effect of Valence (*F*(1,30) = 100.13, MSE = 136.29, *p*<.001), qualified by a Valence×Judgment interaction (*F*(1,30) = 5.03, MSE = 1.81, *p* = .03). Paired samples *t*-tests revealed that intent mattered more for judgments of negative versus positive scenarios in the case of subjective (*t*(30) = −8.77, *p*<.001) and objective (*t*(30) = −9.08, *p*<.001) judgments. We explore this effect of valence in the next experiment.

### 3.4. Art versus Morality

We conducted a Judgment (objective vs. subjective) by Domain (art vs. morality) ANOVA of the absolute values of IA difference scores (for the reasons detailed above) to determine whether intent mattered more for objective versus subjective judgments (across both domains) and whether intent mattered more for moral versus aesthetic judgments (across both objective and subjective judgments). First, this analysis of difference scores revealed a main effect of Judgment (*F*(1,30) = 7.59, MSE = 4.84, *p*<.010); intent mattered more for objective (*M* = 1.63) than subjective judgments (*M* = 1.23), mirroring the patterns observed above for both domains separately.

**Figure 3:**
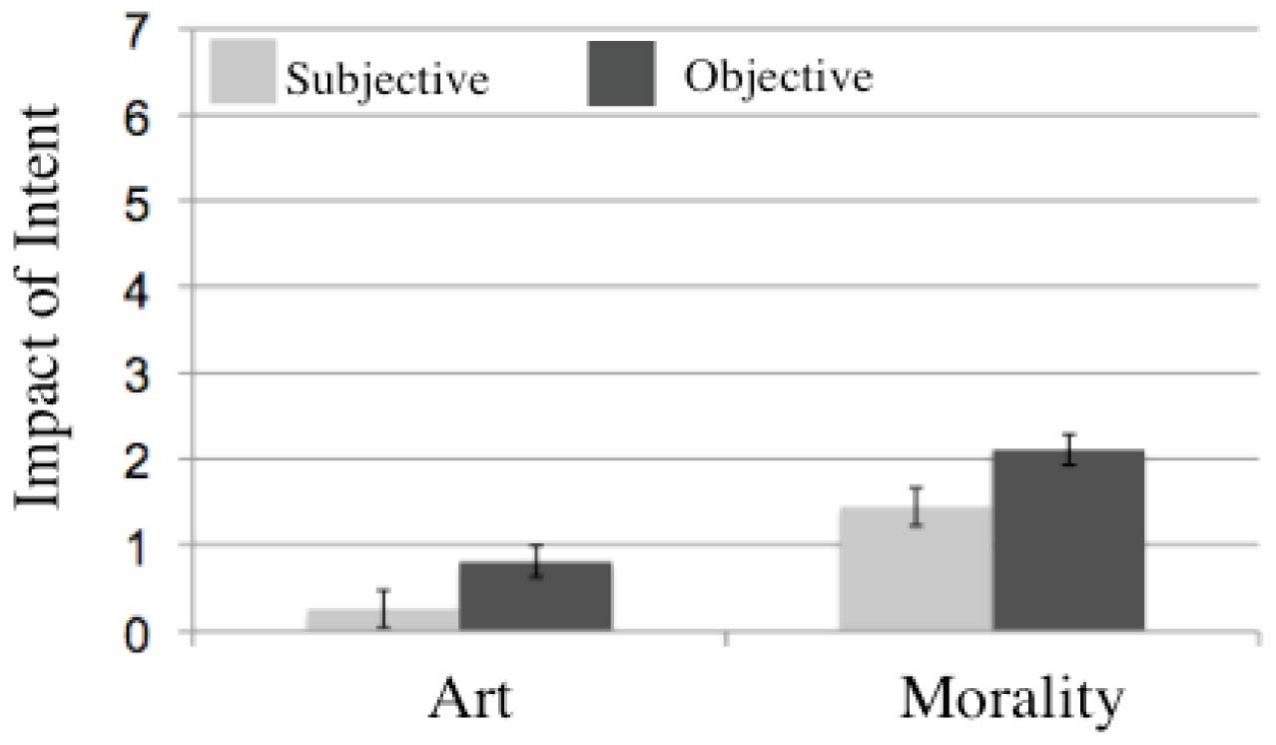
The role of intent in subjective and objective aesthetic and moral judgments. *Y-axis*: IA Difference scores (judgment of intentional act/art minus judgment of accidental act/art) for subjective versus objective judgments, for art and morality. Intent had a greater impact on morality than on art, and on objective than subjective judgments. Figure 3 is a graph of the role of intent in subjective and objective aesthetic and moral judgments. The graph shows the difference scores for subjective vs. objective judgments for art and morality. The graph shows that intent had a greater impact on morality than on art, and on objective than subjective judgments.

Second, as predicted, the same analysis of difference scores revealed a main effect of Domain (*F*(1, 30) = 15.45, MSE = 14.22, *p*<.001); intent mattered more for moral judgments (*M* = 1.77) versus artistic judgments (*M* = 1.09). Finally, a Domain×Judgment interaction (*F*(1,30) = 7.96, MSE = 2.19, *p*<.008) revealed a greater difference in the effect of intent on objective versus subjective judgments in the moral domain (*M*_subjective_ = 1.44, *M*_objective_ = 2.10) versus the art domain (*M*_subjective_ = 1.03, *M*_objective_ = 1.16) (Figure 3).

## Experiment 2

Experiment 2 replicates and extends Experiment 1 by including new images of “bad” art to determine whether the same pattern obtains for “bad” art as for “good” art, and to explore, more generally, a possible effect of valence on the role of intent.

### 4.1. Participants

Participants were 34 new undergraduate psychology majors at Boston College who participated for course credit (13 males, 21 females, ages, 18–21 years, *M* = 19.1).

### 4.2. Materials and Procedure

**Figure 4:**
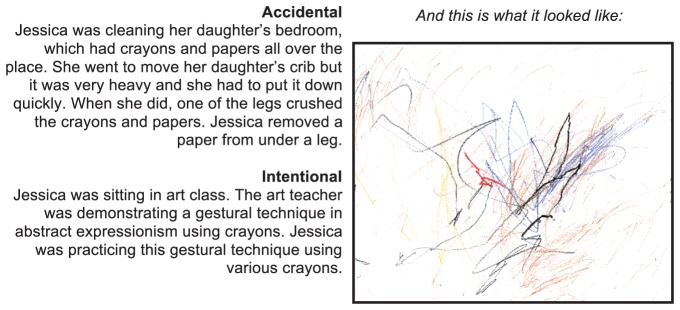
Sample “Bad” Art Image, with narratives. Figure 4 consists of a sample of the stimuli used. The sample is an artwork belonging to the category of “bad” art. The image is accompanied by the intentional and accidental narratives.

Materials and procedure were identical to those outlined in Experiment 1, with the following exceptions. The art stimuli consisted of the images from Experiment 1, plus four “bad” art images, selected from websites displaying art by young children (e.g., scribbles) (Figure 4). In a manipulation check, a paired samples *t*-test revealed that good art (*M* = 4.2) was indeed judged better than bad art (*M* = 1.29) (*t*(33) = 11.285, *p*<.001) (collapsing across subjective and objective judgments).

## Experiment 2 Results

### 5.1. Art

As predicted, paired samples *t*-tests using the absolute values of IA difference scores revealed that intent mattered more for objective than subjective judgments, for good art (*t*(33) = 2.989, *p*<.005) and bad art (*t*(33) = 3.442, *p*<.002). In other words, the intentional versus accidental difference was greater for objective versus subjective judgments of art. Furthermore, participants *liked* accidentally created art (good: *M* = 4.1; bad: *M* = 2.3) but rated it as *less good* objectively (good: *M* = 3.9; bad: *M* = 1.8); this difference between subjective and objective judgments did not emerge for intentionally created art.

### 5.2. Morality

We conducted paired samples *t*-tests using the absolute values of IA difference scores. As in Experiment 1, intent mattered more for objective versus subjective moral judgments, for both positive scenarios (*t*(33) = −3.45, *p*<.002) and negative scenarios (*t*(33) = −2.035, *p*<.05). In other words, the intentional versus accidental difference was greater for objective versus subjective judgments of moral agents.

### 5.3. Art versus Morality

We conducted a Judgment (objective vs. subjective)×Domain (art vs. morality)×Valence (positive/good vs. negative/bad) ANOVA of the absolute values of IA difference scores to determine again whether intent mattered more for objective versus subjective judgments and whether intent mattered more for moral versus aesthetic judgments. We replicated the key patterns found in Experiment 1. First, a main effect of Judgment (*F*(1,33) = 10.85, MSE = 7.28, *p*<.001) revealed intent to matter more for objective (*M* = 1.40) than subjective judgments (*M* = 1.07). Second, a main effect of Domain (*F*(1,33) = 36.24, MSE = 32.141, *p*<.001) revealed intent to matter more for moral judgments (*M* = 1.58) than artistic judgments (*M* = .89). Third, a Domain×Judgment interaction (*F*(1,33) = 4.10, MSE = 3.42, *p*<.05) indicated a greater difference in the impact of intent on objective (*M* = 1.86) versus subjective (*M* = 1.3) judgments in the moral domain versus the art domain (subjective: *M* = .84; objective: *M* = .94).

The same Judgment (objective vs. subjective)×Domain (art vs. morality)×Valence (positive/good vs. negative/bad) ANOVA of the absolute values of IA difference scores revealed, in addition to the primary results above, a main effect of Valence (*F*(1,33) = 32.28, MSE = 22.08, *p*<.001): intent mattered more for judgments of negative items (*M* = 1.52) than positive items (*M* = .95). This main effect was qualified by a Domain×Valence interaction (*F*(1,33) = 49.62, MSE = 61.65, *p*<.001): there was a greater difference between judgments of negative (*M* = 2.34) versus positive *moral* stories (*M* = .82) than between judgments of bad (*M* = .7) versus good *art* (*M* = 1.08). The greater impact of intent on negative versus positive moral judgments is broadly consistent with prior research showing that negative actions elicit greater focus on agents' mental states [34], [18], [35]–[37], as well as recent work showing that people view negative versus positive moral acts through a more objective lens [25].

## General Discussion

The current results support both of our key predictions. First, intent matters more for objective versus subjective judgments not only in the domain of art (Cf. [6]) but also in the domain of morality. Second, intent matters more for moral judgments than for artistic judgments at least in the context of the current study. These results reveal important similarities and differences in the cognitive processes that support our judgments across domains. At the broadest level, people consider the mental states of moral agents and artists when understanding, appreciating, and evaluating their actions. However, as we discuss below, these results also offer a more detailed account of how moral and aesthetic judgments diverge.

### 6.1. Cognitive distinctions common to morality and art

Recent research has explored similar questions for the psychology of morality and the psychology of aesthetics. For example, researchers have investigated whether aesthetic evaluations are influenced primarily by automatic, unconscious, and perhaps emotionally driven processes, or instead evaluative, principled, and controlled processes [38], [15]. In parallel, work in moral psychology and neuroscience has investigated the extent to which “reason” versus “emotion” or “intuition” dominates human moral judgment [39], [40]]–[[44] Literature across both domains has also explored the critical contribution of cultural influences versus universal principles [45], [42].

In keeping with the tensions brought out in this literature on the distinct roles of controlled processing, on the one hand, and automatic processing, on the other, the current study reveals a distinction between “objective” and “subjective” judgments in the domains of art [6] and morality alike. We also reiterate that while evaluating how “good” an entity is may reflect a subjective evaluation in some cases (e.g., “how good is the pie”), our results suggest that, in the present paradigm, participants effectively distinguished between objective evaluations (e.g., how good is the artwork/agent) and subjective evaluations (e.g., do you like the artwork/agent). In particular, participants weighed intent more heavily for objective than subjective evaluations consistently—for both the domain of art [6] and the domain of morality. In other words, objective and subjective judgments as elicited by these questions were in fact associated with distinct cognitive signatures (Cf. [6]).

### 6.2. Cognitive differences between morality and art

As predicted, critical differences also emerged between art and morality in the current work. We observed a significantly greater role for intent in moral versus artistic judgments. Thus, although intent plays a key role in both domains, people may assign more weight to the intent of the moral agent versus the artist. One possible interpretation of the current findings, as presented in the introduction, is that moral judgment, on the whole, is perceived to be more “objective” than aesthetic judgment, even when people are simply evaluating whether they like or dislike an agent for a specific action (a “subjective” judgment in the present paradigm). One can perhaps imagine befriending someone with different taste in art, but not in people [18]. Indeed, according to recent research, robust moral judgments involve not only judgments of the permissibility of an agent's actions but also critical assessments of the agent's character (e.g., [2], [46]. Often, such moral judgments (e.g., do I like this person, would he make a good friend) target whether an agent would make a trustworthy social partner [47]. Nevertheless, prior work reveals important individual differences in such attitudes: some people treat moral beliefs more like objective facts, while others treat moral values more like subjective preferences, [25], [27], [28], [48] Other recent work suggests that meta-ethical attitudes, moral objectivism versus subjectivism, can be primed, with distinct consequences on moral behavior [49]. Future investigations should explore whether moral objectivists assign more weight to intent in general.

Our own ongoing work on meta-ethical intuitions suggests that people are more likely to take a subjectivist approach to art and an objectivist approach to morality. That is, people appear more willing to “agree to disagree” with a confederate who delivers an opposing judgment of an artwork, compared to a confederate who delivers an opposing judgment of a moral action. This pattern may reflect a general tendency to treat artistic judgments like subjective preferences and moral judgment like objective facts, consistent with the present findings (but see [31]]–[[33] for discussion on the objective nature of aesthetic judgments). In addition, not only are people more likely to recognize that opposing artistic versus moral views may be also be right, but also people are more likely to report willingness to befriend the confederate in the case of artistic disagreement versus moral disagreement. These preliminary findings support the overarching claim that the domain of morality may be treated as more objective than the domain of art—again consistent with the current pattern of results that intent matters more for both (1) objective versus subjective judgments and (2) moral versus artistic judgments.

Finally, these results may also relate to recent research suggesting that intent matters more for certain kinds of moral judgments over others [36]. In particular, intent matters more for moral judgments of interpersonally harmful actions (e.g., one person poisons another), compared to moral judgments of purity violations or cultural taboos, which people find offensive even in the absence of objective adverse consequences (e.g., unusual but harmless sexual practices; eating taboo but nutritious foods). Moral norms against purity offenses or taboos may reflect people's subjective preferences or moral *taste* (e.g., what offends people's sensibilities) and therefore reflect a reduced role for intent.

### 6.3. Conclusions

In sum, the present research reveals that intent informs and influences our moral and aesthetic judgments. Recent work has shown that evaluative judgments (e.g., attributions of moral blame) in turn influence mental state inferences, including attributions of intent [34], [37]. Interestingly, convergent research suggests that people may also use the formal properties of an image to reason “backwards” to the intent of the artist [6]. Future work should compare these bi-directional effects for both domains. Of course, mental states fulfil many everyday social functions, not simply moral or artistic evaluation but also predicting and explaining other people's behavior [50], [51]. The possibility that the current approach can be applied to other key domains of social interaction will be worth exploring.

## Supporting Information

Text S1 **A full list of the moral and art scenarios (both positive and negative).**
(PDF)
