# Supplementary material for: Whose Mind Matters More—The Agent or the Artist? An Investigation of Ethical and Aesthetic Evaluations
Source: PLoS One. 2013 Sep 11;8(9):e70759. doi: 10.1371/journal.pone.0070759 (PMC3770669; doi:10.1371/journal.pone.0070759)

Text S1.

Moral Scenarios

Negative

1. When you get to work on Monday morning, you see your co-worker standing outside of your office carrying a new printer. You go over to greet her and see if she needs help, but as you do so, she drops the printer on your foot and breaks one of your toes.

> Accidental: **She jumps back in surprise and you can see that she looks very pained.**
>
> Intentional: **She leans forward and you can see that she's giving you a dirty look.**

2. You and one of your closest friends get into a disagreement. By the time you get home you are feeling upset that you didn't work out your fight in person. When you go to check your email, though, you see that your friend has sent you an email, the subject of which is "I'm sorry".

> Accidental: **You open up the email, to find a long apology. However, your friend has no idea that his email also contains a computer virus that sends dirty pictures to everyone in your address book. You are completely mortified.**
>
> Intentional: **You open up the email, only to find that your friend is the opposite of sorry—he has intentionally sent you a computer virus that sends dirty pictures to everyone in your address book. You are completely mortified.**

3. You and your sister have recently gotten into a fight. You've forgiven your sister, and you think that she has forgiven you. She asks to borrow your car to go pick up a friend up. When she returns your car, you see that she spilled nail polish all over the seats.

> Accidental: **It turns out this was a complete accident. Your sister is incredibly apologetic.**
>
> Intentional: **It turns out she did this on purpose because she was still feeling very angry at you.**

Positive

Whose Mind Matters More—Text S1

1. Your cousin breeds dogs for a living. In his newest litter of golden retrievers, there is one puppy left.

> Accidental: **Nobody has chosen that puppy, so your cousin asks whether you'll take that puppy home. He has no idea that all your life you have wanted a golden retriever. You go home happy, with your new puppy.**
>
> Intentional: **Your cousin has been saving a puppy for you. He asks whether you'll take that puppy home because he knows that all your life you have wanted a golden retriever. You go home happy, with your new puppy.**

2. You have been home sick for the past few days and feeling lonely. But then one of your classmates stops by your apartment.

> Accidental: **Your teacher had asked him to stop by with some homework on his way to class. You are thrilled to see a familiar face. Your classmate tells you about what you've missed before heading back to campus.**
>
> Intentional: **He heard you were stuck at home sick and stops to visit on his way to class. You are thrilled to see a familiar face. Your classmate tells you about what you've missed before heading back to campus.**

3. One of your colleagues comes over to your house to drop off a book he borrowed from you.

> Accidental: **He's carrying a stack of other books that he's about to get rid of at a yard sale. In the stack you see a book you've been trying to find for years. Your colleague has no idea how much this book means to you. He gives you the book and is surprised by how happy you are.**
>
> Intentional: **He's carrying a stack of other books that he's about to get rid of at a yard sale. But he's also brought you a book you've been trying to find for years. In fact he tracked it down at an old bookstore just for you. He gives you the book and is pleased by how happy you are.**

Art Scenarios

Good Art

1. Sally was painting her bedroom:

Accidental: **She wanted to paint one wall green and one wall with red and black flowers. She ended up picking up the wrong paintbrush and mixing the colors.**

Intentional: **She was using specific color patterns and a deliberate method of laying down the paint. She carefully blended and separated her brushstrokes on the wall.**

This is what it looked like:

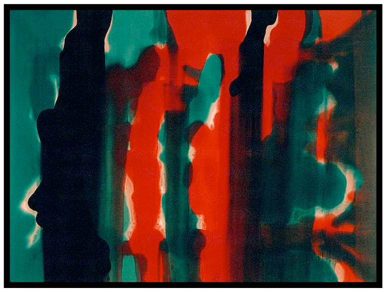

2. James:

Accidental: **was painting his bathroom. After he was done painting he realized that he had spilled some paint from the cans he was using. He tried cleaning it up with a sponge, but it was mostly dry.**

Intentional: **was working on a new project using sponges. He dipped the sponges in paint and then carefully laid the paint on the canvas making various shapes. He organized the space in terms of the color he used and the amount of paint he laid down.**

This is what it looked like:

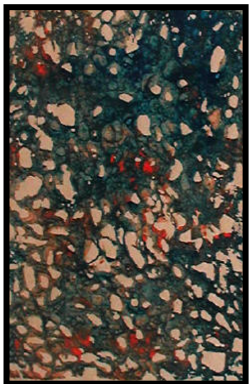

3. Laura:

Accidental: **was waiting for her friend to come over. She saw that her sister left out paints. She started doodling random designs without looking at what she was doing.**

Intentional: **sat down with her paints one day. She took her paintbrush and decided to make a pink flower. She organized the composition in an abstract way.**

This is what it looked like:

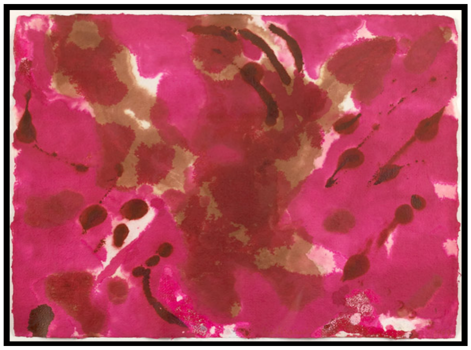

Bad Art

1. Max was asked to paint a mural for his local elementary school:

Accidental: **He took all the colors he had and scribbled these patches of all the different colors he could use.**

Intentional: **He decided to make a representational piece, symbolizing the unity of students from all backgrounds and of all colors.**

This is what it looked like:

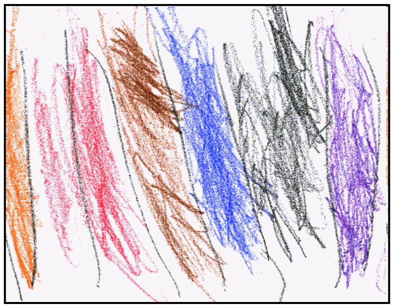

2. Linda was using a calligraphy brush. She made a few simple lines with it:

Accidental: **But then as she took the brush out of the ink, it dripped messy lines all over the left hand side of the paper.**

Intentional: **Then she made a Roman inspired calligraphic tree on the left hand side of the paper.**

This is what it looked like:

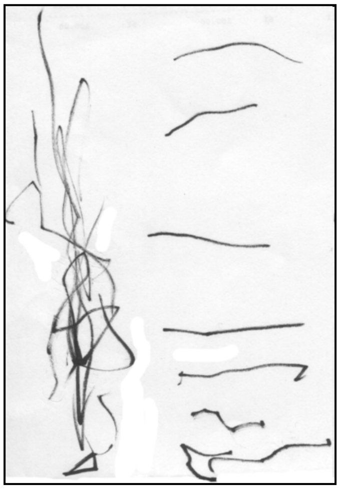

3. John was working on a project for school.

Accidental: **He got out his markers and started testing them on a sheet of paper. He tested a few different colors.**

Intentional: **He got out his markers and started using them. He was using a technique he learned called "push and pull" in which you put brighter colors in the foreground.**

This is what it looked like:

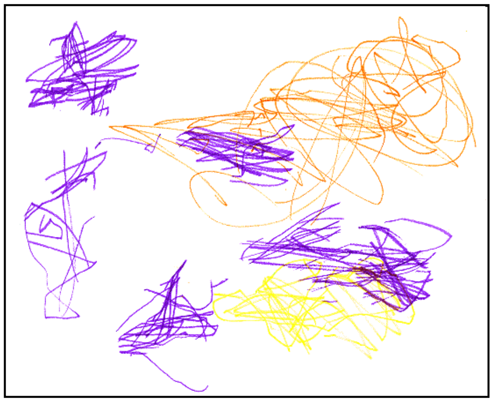

Supplement: Text S1 — A full list of the moral and art scenarios (both positive and negative). (PDF) [file pone.0070759.s001.pdf]
